# Cancer Preventive Efficacy of Marine Carotenoid Fucoxanthin: Cell Cycle Arrest and Apoptosis

**DOI:** 10.3390/nu5124978

**Published:** 2013-12-06

**Authors:** Thamaraiselvan Rengarajan, Peramaiyan Rajendran, Natarajan Nandakumar, Maruthaiveeran Periyasamy Balasubramanian, Ikuo Nishigaki

**Affiliations:** 1 NPO-International Laboratory of Biochemistry, 1-166, Uchide, Nakagawa-ku, Nagoya 454-0926, Japan; E-Mails: thamarairaj2000@gmail.com (T.R.); peramaiyanrajendran@gmail.com (P.R.); 2 Department of Microbiology, Immunology and Genetics, Ben Gurion University of the Negev, Beer Sheva 84105, Israel; E-Mail: rajnandakumar@yahoo.com; 3 Department of Pharmacology and Environmental Toxicology, Dr. A. L. Mudaliar Post Graduate Institute of Basic Medical Sciences, University of Madras, Taramani Campus, Chennai 600113, India; E-Mail: balumppet@gmail.com

**Keywords:** cancer, fucoxanthin, apoptosis, cell cycle, signaling

## Abstract

Epidemiological investigations have shown that overcoming the risk of cancer is related to the consumption of green vegetables and fruits. Many compounds from different origins, such as terrestrial plants and marine and microbial sources, have been reported to have therapeutic effects of which marine sources are the most important because the diversity of marine life is more varied than other sources. Fucoxanthin is one important compound with a marine origin and belongs to the group of carotenoids; it can be found in marine brown seaweeds, macroalgae, and diatoms, all of which have remarkable biological properties. Numerous studies have shown that fucoxanthin has considerable medicinal potential and promising applications in human health. In this review, we summarize the anticancer effects of fucoxanthin through several different mechanisms including anti-proliferation, induction of apoptosis, cell cycle arrest and anti-angiogenesis, and its possible role in the treatment of cancer.

## 1. Introduction

**Figure 1:**
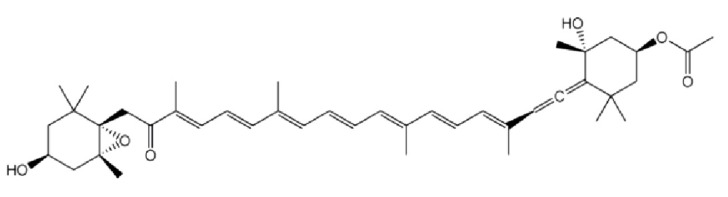
Structure of Fucoxanthin.

The incidence of cancer and the failure of conventional chemotherapy to achieve a reduction in the mortality rates for common epithelial malignancies such as carcinomas of the lung, colon, breast, prostate and pancreas indicate a critical need for new approaches to control cancer development [1,2]. Chemoprevention is a novel and promising approach to control, inhibit or suppress tumor cell proliferation. Chemoprevention has been successfully achieved in numerous *in vitro* and *in vivo* studies and has been validated in several human intervention trials [3]. Many population-based studies have highlighted the ability of macronutrients and micronutrients in vegetables and fruits to reduce the risk of cancer. Recently, attention has been focused on phytochemicals, non-nutritive components in a plant-based diet that possess cancer-preventive properties [4]. Many clinical trials on the use of nutritional supplements and modified diets to prevent cancer are ongoing. It is conceivable that, in the future, people might only need to take specially formulated pills that contain substances derived from edible plants to prevent cancer or delay its onset [5]. However, a precise assessment of the mechanisms by which the components of fruits and vegetables prevent cancer is necessary before they can be recommended for inclusion in dietary supplements or before they can be tested in human intervention trials. Phytonutrients that include the yellow, orange and red carotenoid pigments have recently been investigated. Epidemiologically, the intake of dietary carotenoid from fruit and vegetable sources has been correlated with a reduced cancer risk [6,7,8]. Fucoxanthin is one of the most abundant carotenoids and contributes to more than 10% of the estimated total production of carotenoids in nature, especially in the marine environment [9]. Fucoxanthin is a pigment that, along with chlorophylls and β-carotene, is widely distributed in brown algae and diatoms [10,11]. It has an unusual structure that includes an allenic bond and a 5,6-monoepoxide moiety (Figure 1). *Undaria pinnatifida*, the brown seaweed better known as wakame, is a rich source of fucoxanthin [12]. *U. pinnatifida* is widely used as a human food in many countries, especially Korea and Japan, and it is becoming increasingly popular in the European market, mostly in the form of extracts used as food supplements. The numerous activities attributed to *U. pinnatifida* products are essentially linked to their fucoxanthin content [13,14].

Fucoxanthin is a xanthophyll with antioxidant properties [15] whose distinct structure includes an unusual allenic bond, an epoxide group and a conjugated carbonyl group in a polyene chain [16]. Japanese researchers have performed single and repeated toxicity studies of fucoxanthin to evaluate the safety of this carotenoid. In a single dose study, fucoxanthin was administered orally to male and female mice at the dosages of 1000 and 2000 mg/kg, and in the repeated doses study, fucoxanthin at the dosages of 500 and 1000 mg/kg was administered orally for 30 days; in both studies, there were no fatalities or abnormalities reported, and the gross appearance of the mice remained unchanged. In another study, the repeated doses of fucoxanthin resulted in normal histology and no abnormal changes in the liver, kidney, spleen and gonadal tissues of any of the fucoxanthin-treated groups [13,17]. Subsequently, Beppu *et al.* [17] found that fucoxanthin did not have a genotoxic/mutagenic effect on the bone marrow cells of mice. Iio *et al.* [18] investigated the subchronic toxicity of fucoxanthin in rats and the genotoxicity in mice and found that there was no morbidity in single oral dose of fucoxanthin and the 50% lethal dose of fucoxanthin was more than 2000 mg/kg body weight. In another investigation, a 13-week oral dose study showed that there were no adverse effects of fucoxanthin at a dose of 200 mg/kg body weight under the subchronic dose conditions. These studies suggested that fucoxanthin was a safe compound and did not exhibit toxicity and mutagenicity under these experimental conditions [19,20]. Numerous reports on the protective effect of fucoxanthin against drug toxicity suggest a possible complementary role for fucoxanthin in improving the quality of life of cancer patients. The potential of Fucoxanthin has led scientists to investigate its molecular mechanism(s) and evaluate its significance in the treatment of cancer. Further, numerous *in vitro* and *in vivo* studies have provided ample evidence that Fucoxanthin could prevent carcinogenesis and inhibit tumorigenesis through different molecular mechanisms. Hence, the focus of this review is to discuss the molecular targets modulated by fucoxanthin and its potential therapeutic implications in cancer.

## 2. Anti Proliferative Potential of Fucoxanthin

Studies have shown that fucoxanthin exhibits anti-proliferative potential in different types of carcinomas including prostate cancer (PC-3, DU145, LNCaP), leukemia (HL-60, HP50-2, HP100-1, ATL), colon cancer (HT-29, caco-2, DLT-1, LS1174T), liver cancer (HepG2, S-Hep-1), urinary bladder cancer (EJ-1), gastric cancer (MGC-803), breast cancer (MCF-7), melanoma (B16F10) and lymphoma (PEL) in a dose dependent manner [21,22,23,24,25,26,27,28,29]. The antitumor effect of fucoxanthin has been shown to be mediated through the up-regulation of the p21WAF1/Cip1, ROS-mediated Bcl-xl pathway, the down-regulation of the cyclin D, JAK/STAT (Janus Kinase/Signal Transducer and Activator of Transcription) signal pathway and is associated with GADD45a, p38 MAPK and SAPK/JNK [22,26,30,31,32,33]. Fucoxanthin significantly inhibited the formation and development of aberrant crypt foci, a preneoplastic marker for colon cancer, in mice induced by azoxymethane and 1,2-dimethylhydrazine dihydrochloride [34,35]. Fucoxanthin has been proven to suppress spontaneous liver tumorigenesis in C3H/He male mice and has shown antitumor-promoting activity in a two-stage experimental carcinogenesis initiated with 7,12-dimethylbenz[*a*]anthracene and promoted with 12-*O*-tetradecanoylphorbol-13-acetate and mezerein in the skin of ICR mice [36]. In addition, fucoxanthin was reported to inhibit duodenal carcinogenesis induced by *N*-ethyl-*N*′-nitro-*N*-nitrosoguanidine in mice [37]. These results suggest that fucoxanthin may be useful in treating different types of malignancies.

## 3. Fucoxanthin Induces Apoptosis

**Figure 2:**
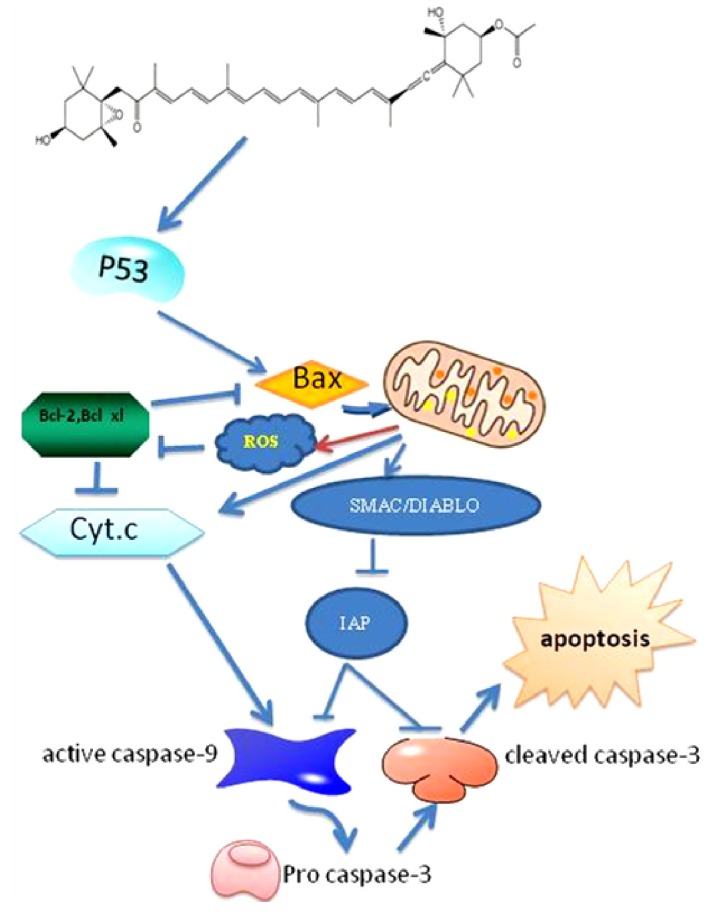
Fucoxanthin and the apoptosis signaling pathway.

Apoptosis is the selective process of physiological cell deletion that regulates the balance between cell proliferation and cell death. The failure of apoptosis is thought to contribute generally to the development of human malignancies. The induction of apoptosis is now considered to be an attractive strategy for cancer therapy [38,39]. The caspases play a central role in the extrinsic and intrinsic pathways of apoptosis [25,40]. One of the apoptosis-inducing pathways is mediated by the release of cytochrome c from the mitochondria to the cytosol, followed by the activation of caspase-9 and -3. The activated caspase-3 is capable of cleaving many cellular substrates, including inhibitors of caspase-activated DNase (ICAD), Poly ADP-Ribose Polymerase (PARP; a DNA repair enzyme), and lamin. Once the ICAD is cleaved, CAD (caspase-activated DNase) enters the nucleus and breaks the chromatin into DNA fragments. Subsequent disassembly of the cell structure eventually leads to cell death [25,41,42]. Bcl-2 and Bcl-xL are upstream molecules in the apoptotic pathway and are identified as potent suppressors of apoptosis [43,44]. Kim *et al.* [22], reported that fucoxanthin induced the cleavage of caspases-3 and -7 and poly-ADP-ribose polymerase (PARP) and decreased Bcl-xL levels, whereas NAC (*N*-acetylcysteine) pre-treatment significantly inhibited the cleavage of caspases-3 and -7 and PARP and the reduction in Bcl-xL levels. Fucoxanthin generated ROS (Reactive Oxygen species), and the accumulation of ROS performed a crucial role in the fucoxanthin-induced Bcl-xL signaling pathway. Zhang *et al.* [25] showed that fucoxanthin significantly reduced the viability of the urinary bladder cancer EJ-1 cell line in a dose- and time-dependent manner. The induction of apoptosis in EJ-1 cells was characterized by morphological changes, a DNA ladder, and an increased percentage of hypodiploid cells activating caspase-3 activity. Kotake-Nara *et al.* [45] showed that apoptosis induced by fucoxanthin in HL-60 cells was associated with a loss of mitochondrial membrane potential at an early stage but not with an increase in reactive oxygen species. Fucoxanthin treatment caused the cleavage of procaspases-3 and poly-ADP-ribose polymerase (PARP) without any effect on the protein levels of Bcl-2 and Bax or the induction of apoptosis mediated by mitochondrial membrane permeabilization and caspase-3 activation [29]. Furthermore, fucoxanthin induces DNA fragmentation, which is a hallmark of apoptosis, in HL-60 cells [46]. Yamamoto *et al.* [47] assayed both *in vivo* and *in vitro* models of primary effusion lymphoma (PEL) and found that fucoxanthin effectively induced apoptosis by the down-regulation of anti-apoptotic proteins and the up-regulation of the caspase pathway. Furthermore, similar findings were reported in Sarcoma 180 (S180) Xenografts-Bearing Mice.In addition, fucoxanthin induces apoptosis in colon cancer cell lines such as Caco-2, HT-29 and DLD-1 [23]. Januar *et al.* [48] showed fucoxanthin activate the p53 expression. Fucoxanthin-induced apoptosis was accompanied by the down-regulation of the protein levels of Bcl-xL, resulting in a sequential activation of caspase-9, caspase-3, and PARP *in vivo* in Balb/c mice [28]. The inhibitor-of-apoptosis (IAP) proteins suppress cell death by inhibiting the activity of caspases; this inhibition is performed by the zinc-binding BIR (Baculovirus IAP Repeat) domains of the IAP proteins. The mitochondrial protein Smac/DIABLO promotes apoptosis by eliminating the inhibitory effect of IAPs through physical interactions [48]. XIAP, cIAP1 and cIAP2 are direct inhibitors of at least two members of the caspase family of cell death proteases: caspase-3 and caspase-9 [49]. Ishikawa *et al.* [31] evaluated the effects of fucoxanthin and its metabolite fucoxanthinol on adult T-cell leukemia (ATL). Both carotenoids induced apoptosis in HTLV-1-infected T-cell lines, which was associated with the activation of caspases-3, -8 and -9, as well as down-regulation of the expression of the anti-apoptotic proteins, XIAP, cIAP2, Bcl-2 and survivin; XIAP and cIAP2 inhibit caspase-3 and 9 activities, respectively [31] (Figure 2). Because survivin can inhibit caspase-3, it is possible that the down-regulation of survivin by the two carotenoids could lead to the activation of caspase-3 [34]. The results suggested that the anticancer activity of fucoxanthin depends on the cell type.

## 4. Cell Cycle Arrest

**Figure 3:**
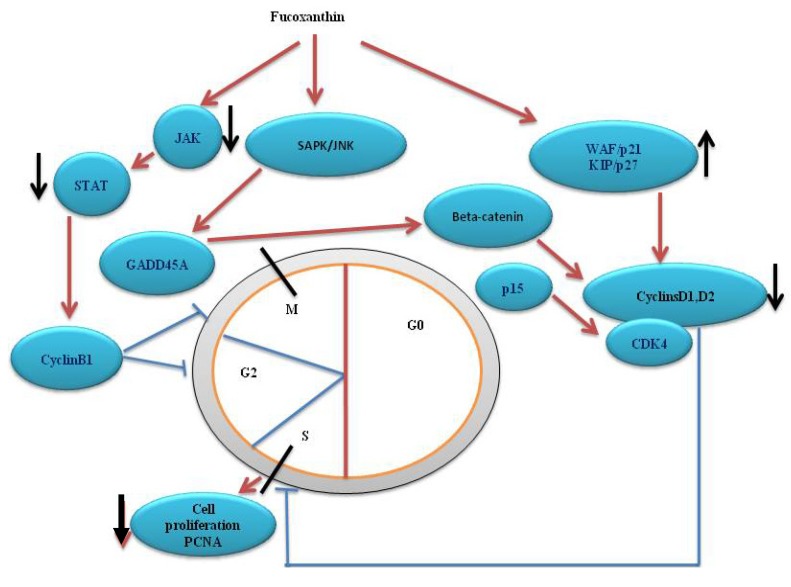
Fucoxanthin on cell cycle arrest. ↑ Up regulation; ↓ down regulation.

The growth of cells is normally determined by the extracellular signals that control the gene expression and protein regulation required for cell division [50]. In contrast, during tumor progression, cancer cells are conferred with the capacity to proliferate independently of exogenous growth-promoting or growth-inhibitory signals [30,51,52]. Thus, the anti-proliferative effect of chemicals or drugs on cancer cells is one of the mechanistic ways to exert their anti-carcinogenic activity. Fucoxanthin has shown its anti-neoplastic effects in various cell lines by inducing cell cycle arrest. Yu *et al.* [26] reported that fucoxanthin effectively induced cell cycle arrest in the G2/M phase and apoptosis in gastric cancer MGC-803 cells and that the down-regulation of survivin and CyclinB1 might contribute to the anti-cancer effects of fucoxanthin. Fucoxanthin may reduce CyclinB1 expression through the JAK/STAT signal pathway, sequentially inhibiting the proliferation of MGC-803 cells. Das *et al.* [53] reported that the expression of the cyclin dependent kinase inhibitor p21Waf1/Cip1 was not influenced by fucoxanthin treatment in HepG2 cells. In contrast, Satomi *et al.* [54] have previously found that fucoxanthin induced G1 arrest in HepG2 and DU145 cells. The induction of GADD45a seems to be associated with the G1 arrest induced by fucoxanthin. Fucoxanthin also induced PIM1 and GADD153in both cell lines, while *CYP1A1* expression was markedly increased in only the HepG2 cells. They hypothesized that GADD45a, a p53-regulated gene interacting with the products of two different p53-regulated genes, p21Waf1/Cip1 and proliferating cell nuclear antigen, are involved in fucoxanthin-induced G0/G1 arrest [55,56]. Ji *et al.* [57] demonstrated that in MEF cells, GADD45a induction is followed by cyclin D1 suppression by inhibiting the translocation of β-catenin to the nucleus. Fucoxanthin has been reported to be involved in the decreased expression of cyclin D1, which may be driven by changes in GADD45a expression in HepG2 cells [54]. Recently, Kim *et al.* [28] found that fucoxanthin has anti-proliferative effects on B16F10 cells through the induction of apoptosis and cell-cycle arrest. It increased the proportion of cells in the G_1_ phase of the cell cycle, which was associated with decreased cyclin D1 and D2, and CDK4 expression and increased p15INK4B and p27Kip1 expression. Moreover, fucoxanthin induces G1 cell cycle arrest through a GADD45a-dependent pathway.Fucoxanthin-induced GADD45a expression and G_1_ arrest are negatively regulated by the p38 MAPK pathway in HepG2 cells and positively regulated by the SAPK/JNK pathway in DU145 cells. Additionally, Satomi and Nishino suggested that GADD45a is involved in the G_1_ arrest induced by fucoxanthin and that the pattern of MAPK involvement in the induction of GADD45a and G_1_ arrest by fucoxanthin changes its anti-proliferative effect depending on the cell type [32] (Figure 3). Together, these results indicate that cell cycle arrest is likely to be one of the important mechanisms for the anticancer activity of fucoxanthin.

## 5. Fucoxanthin on Signaling Pathway

The JAK/STAT pathway is a conserved signal transduction pathway that has been implicated in a number of distinct developmental and disease processes. Cytokines mediate their responses through the activation of the JAK/STAT signaling pathway, STATs comprising a family of seven structurally and functionally related proteins and JAKs representing a family of four non-receptor tyrosine kinases, which selectively phosphorylate and therefore activate STATs [58]. The activated STAT plays a critical role in regulating the innate and acquired host immune responses. Of these STATs, STAT3 plays an important role during the activation of the JAK/STAT pathway, while AG490 (tyrphosin AG490, inhibitor of JAK kinase) can effectively block the JAK/STAT pathway [26,59]. Yu *et al.* [26] reported that fucoxanthin reduced the expression of STAT3 at the mRNA and protein level suggesting that fucoxanthin inhibits the JAK/STAT pathway. In the presence of AG490, the expression of p-STAT3 and survivin were also reduced to a level slightly lower than that of the vehicle control group. With the co-treatment of fucoxanthin and AG490, the reduced expression of STAT3, p-STAT3 and CyclinB1 by fucoxanthin was inhibited [26]. Recently, Liu *et al.* [60] used specific inhibitors to show that fucoxanthin inhibited ERCC1 mRNA expression through the ERK and PI3K/AKT pathways, whereas this carotenoid inhibited TP (thymidine phosphorylase) expression through the p38 pathway. The results indicate that the improved chemotherapeutic efficacy of fucoxanthin may also involve the inhibition of the mRNA expression of some DNA repair genes through the down-regulation of the ERK, p38, and PI3K/AKT pathways. The role of JAK/STAT in the anti-cancer effect of fucoxanthin is still not well understood, and further study is warranted to explain the significance of this pathway.

The NF-κB pathway is known to suppress apoptosis through the loss of pro-apoptotic factors (e.g., functional p53 or Bax) or the activation of anti-apoptotic factors such as Bcl-2, Bcl-XL or IAPs [61]. Moreover, NF-κB binding sites were found in the promoter of Bcl-2, Bcl-xL, and survivin suggesting that these apoptotic factors may be regulated by NF-κB. Over-expression of the anti-apoptotic molecules Bcl-2 or Bcl-xL can cause resistance to anticancer drugs [60,62,63]. Liu *et al.* [61] showed that fucoxanthin pretreatment attenuated the cisplatin-induced DNA-binding activity of NF-κB, restored cisplatin-inhibited IκB-α***-***phosphorylation, and increased the ratio of Bax/Bcl-2 mRNA expression rendering cancer cells sensitive to apoptosis induced by cisplatin, which suggested that fucoxanthin may be an NF-κB inhibitor. The effects of fucoxanthin may involve the inhibition of NF-κB expression and an increase in the Bax/Bcl-2 mRNA ratios regulated by NFκB. Along with fucoxanthin, fucoxanthinol also suppressed IkBa phosphorylation and JunD expression, resulting in the inactivation of nuclear factor-κB and activator protein-1 molecules. Ishikawa *et al.* [31] found that the suppression of NF-κB by fucoxanthin and fucoxanthinol correlated with the down-regulation of the expression of several gene products regulated by NF-κB. They inhibited IκB-α phosphorylation and NF-κB DNA-binding activity.

## 6. Anti-Angiogenic Effect

The discovery of natural compounds as effective angiogenesis inhibitors has become an important approach in the prevention of cancer. In this context, Sugawara *et al.* [64] investigated the anti-angiogenic effects of fucoxanthin using cultured human umbilical vein endothelial cells and the rat aortic ring and found that fucoxanthin could significantly suppress the differentiation of endothelial progenitor cells into endothelial cells and the formation of new blood vessels, as well as significantly reduce the tube length of endothelial cells. These results clearly showed that fucoxanthin and fucoxanthinol inhibited microvessel outgrowth in an *ex vivo* angiogenesis assay using a rat aortic ring, which suggests that the anti-angiogenic effect of fucoxanthin might be useful in preventing angiogenesis-related diseases, such as cancer, diabetic retinopathy, atherosclerosis and psoriasis [64]. Wang *et al.* [47] showed that fucoxanthin significantly decreased the expression of vascular endothelial growth factor (VEGF) in Sarcoma 180 (S180) of Xenografts-Bearing Mice. Recently, Ganesan *et al.* [65] demonstrated for the first time that the molecular mechanism underlying the anti-angiogenic effects of fucoxanthin and siphonaxanthin is the down-regulation of signal transduction by fibroblast growth factor 2 (FGF-2) and its receptor (FGFR-1). Therefore, these investigations clearly showed that fucoxanthin has promising effects against cancer angiogenesis, but more in depth studies are needed to explain the molecular mechanism(s) involved.

## 7. Conclusions

Fucoxanthin has preventive effects on cancer through different mechanisms of action. Numerous studies have shown that the different mechanisms of anticancer action include anti-proliferation, cell cycle arrest, apoptosis induction, suppression of angiogenesis and antidrug potential. Moreover, fucoxanthin could attenuate the toxicity associated with the use of conventional medicine without compromising its therapeutic efficacy. The various molecular targets of fucoxanthin have been identified using various cancer cell lines; however, further research in animal models of disease is warranted to obtain more conclusive evidence for the molecular basis of fucoxanthin action. Despite its demonstrated therapeutic efficacy in tumor cell lines and animal models, there have been few clinical studies using fucoxanthin to date. Moreover, data on the bioavailability and other pharmacokinetic parameters of fucoxanthin are still incomplete. More studies need to be performed systematically before fucoxanthin can be developed into a drug for the potential treatment of various carcinomas.
